# Ara h 2 is the dominant peanut allergen despite similarities with Ara h 6

**DOI:** 10.1016/j.jaci.2020.03.026

**Published:** 2020-09

**Authors:** Oliver Hemmings, George Du Toit, Suzana Radulovic, Gideon Lack, Alexandra F. Santos

**Affiliations:** a Department of Women and Children’s Health (Pediatric Allergy), School of Life Course Sciences, Faculty of Life Sciences and Medicine, King’s College London, London, United Kingdom; b Peter Gorer Department of Immunobiology, School of Immunology and Microbial Sciences, Faculty of Life Sciences and Medicine, King’s College London, London, United Kingdom; c Asthma UK Centre in Allergic Mechanisms of Asthma, London, United Kingdom; d Children’s Allergy Service, Evelina London Children's Hospital, Guy’s and St Thomas’ Hospital, London, United Kingdom

**Keywords:** Ara h 2, Ara h 6, specific IgE, mast cell activation test, ImmunoCAP inhibition, peanut allergy, cross-reactivity, AIT, Allergen-specific immunotherapy, Ara h, *Arachis hypogaea*, EC50, Half maximal effective concentration, MAT, Mast cell activation test, OFC, Oral food challenge, ROC, Receiver operating characteristic, SPT, Skin prick test

## Abstract

**Background:**

*Arachis hypogaea* 2 (Ara h 2)-specific IgE is to date the best serologic marker to diagnose peanut allergy. Ara h 6 shares approximately 60% sequence identity and multiple epitopes with Ara h 2.

**Objective:**

Our aim was to assess the diagnostic utility and relative importance of Ara h 2 and Ara h 6 in peanut allergy.

**Methods:**

A cohort 100 of children was studied. The cohort included chidren who had peanut allergy, children who were sensitized to but tolerant of peanut, and children who were neither sensitized nor allergic to peanut. Levels of specific IgE to peanut and individual allergens were quantified by using ImmunoCAP. ImmunoCAP inhibition experiments and mast cell activation tests in response to both Ara h 2 and Ara h 6 were performed. Statistical analyses were done using SPSS version 14 and Prism version 7 software.

**Results:**

Ara h 2–specific IgE and Ara h 6–specific IgE showed the greatest diagnostic accuracy for peanut allergy when compared with specific IgE to peanut and other peanut allergens. Most patients with peanut allergy were sensitized to both Ara h 2 and Ara h 6. Ara h 2 reduced Ara h 2–specific IgE binding more than Ara h 6 did (*P* < .001), whereas Ara h 6–specific IgE binding was inhibited to a similar degree by Ara h 2 and Ara h 6 (*P* = .432). In the mast cell activation test, Ara h 2 induced significantly greater maximal reactivity (*P* = .001) and a lower half maximal effective concentration (*P* = .002) than did Ara h 6 when testing cosensitized individuals.

**Conclusions:**

Ara h 2–specific IgE and Ara h 6–specific IgE provide the greatest accuracy to diagnose peanut allergy. Ara h 2 is the dominant conglutin in peanut allergy in the United Kingdom, despite a degree of cross-reactivity with Ara h 6.

## Graphical abstract

**Figure:**
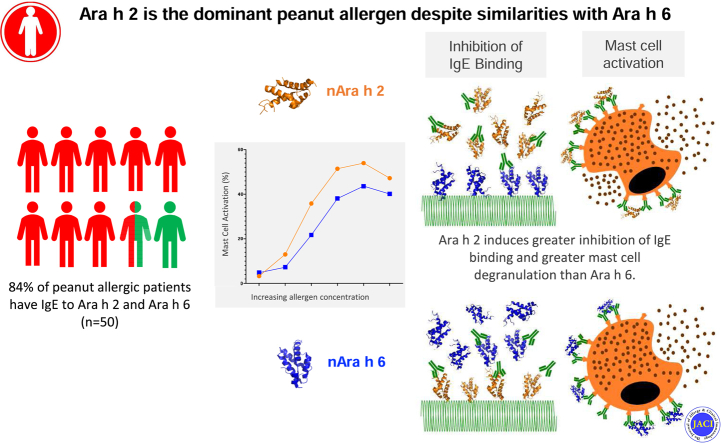

Peanut allergy is a prominent IgE-mediated food allergy that has garnered substantial clinical attention owing to the severity of reactions and the increasing prevalence and rates of anaphylaxis.^1^ Peanut allergy affects about 25% of children with food allergy in the United States. Although many food allergies are transient with age, peanut allergy appears to be particularly persistent.^2^ Because of the systemic nature of peanut allergy, its symptoms can be manifested in several organs, including in the oral, cutaneous, gastrointestinal, cardiovascular, and respiratory systems, potentially leading to fatal reactions.

Detection of allergen-specific IgE supports the diagnosis of peanut allergy; however, sensitization alone is not predictive. In the United Kingdom, 11.8% of school-aged children have detectable levels of peanut–specific IgE; however, only 2.6% of these children are genuinely allergic when challenged.^3^ The utility of allergen-specific IgE assays to diagnose peanut allergy has improved with the analysis of IgE to individual allergen components. A total of 16 peanut allergens have been identified from *Arachis hypogaea* 1 (Ara h 1) to Ara h 17, excluding only Ara h 4, which was initially established with unique nomenclature before being relegated as an Ara h 3 isoform (3.02).^4^^,^^5^ Among peanut components, there are 2 cupins (Ara h 1 and Ara h 3), 3 conglutins (Ara h 2, Ara h 6, and Ara h 7), 1 profilin (Ara h 5), 1 PR-10 protein (Ara h 8), 4 oleosins (Ara h 10, Ara h 11, Ara h 14, and Ara h 15), 3 non-specific lipid transfer proteins (Ara h 9, Ara h 16, and Ara h 17), and 2 defensins (Ara h 12 and Ara h 13).

Ara h 1, Ara h 2, and Ara h 3 were initially identified as the major peanut allergens, with 97% of patients with peanut allergy sensitized to at least 1 of these allergens.^6^ These were also the peanut components that offered greatest discrimination between individuals with and without peanut allergy; in particular, Ara h 2–specific IgE has notably improved the diagnostic accuracy of peanut allergy.^7^^,^^8^ More recently, Ara h 6 has also been identified as a major peanut allergen.^9^, ^10^, ^11^ Both Ara h 2 and Ara h 6 are 2S albumins, have similar molecular weights (17 kDa and 15 kDa respectively), share approximately 60% sequence identity, and are expressed at similar levels across numerous peanut varieties.^11^^,^^12^ The latter fact results in comparable levels of exposure to both Ara h 2 and Ara h 6 when individuals with allergy consume peanuts. Even though only the structure of the protease-resistant core of Ara h 6 has been solved,^13^ it appears that Ara h 6 and Ara h 2 are conformationally similar. Both peanut 2S albumins are resistant to high temperatures and proteolytic digestion, and they are considered the peanut allergens with the greatest ability to induce effector cell activation.^9^^,^^14^^,^^15^ Clinically, Ara h 2 and Ara h 6 sensitization in individuals with peanut allergy is associated with more severe forms of peanut allergy.^16^

Given the sequential and structural similarities between Ara h 2 and Ara h 6, we aimed to assess the relative importance of Ara h 2 and Ara h 6 in peanut allergy, their possible cross-reactivity, and the utility of specific IgE to these allergens to diagnose peanut allergy.

## Methods

### Study population

Individuals who were (1) allergic to peanuts, (2) sensitized to but tolerant of peanut, or (3) neither sensitized nor allergic to peanuts and enrolled into the study “Diagnostic Markers of Clinical Allergy versus Sensitization to Peanut” were evaluated. Peanut allergy was determined by a positive oral food challenge (OFC) result, except in the case of patients who had a convincing clinical history of systemic allergic reactions within 1 year of sample drawing, together with a skin prick test (SPT) wheal size of 8 mm or greater and/or a peanut-specific IgE titer of 15 kU_A_/L or greater.^17^ Peanut tolerance was defined by a negative OFC result or the ability to ingest 4 g or more of peanut protein twice a week without demonstrating an allergic response, as monitored by a validated peanut consumption questionnaire.^18^ Peanut sensitization was determined by an SPT wheal size of 1 mm or greater and/or a peanut-specific IgE titer of 0.1 kU_A_/L or greater. A total of 100 patients were selected purely on the basis of availability of serum samples of sufficient volume. The study and the use of samples were approved by the South East London Research Ethics Committee 2, and written informed consent was obtained from the parents of all of the children.

### Allergen-specific IgE measurements

Levels of specific IgE to peanut and peanut components Ara h 1, Ara h 2, Ara h 3, Ara h 6, Ara h 8, and Ara h 9 were quantified in patient plasma by using the ImmunoCAP assay (Thermo Fisher, Uppsala, Sweden) and the Phadia 100 analyzer according to the manufacturer’s instructions. Quantification of IgE to Ara h 1, Ara h 2, Ara h 3, Ara h 8, and Ara h 9 was performed at the time of recruitment and IgE to Ara h 6 at a later time, when the test became commercially available. Any values that indicated a specific IgE level greater than 100 kU_A_/L were diluted with specific IgE diluent (Thermo Fisher) 1 in 10 and retested.

The ImmunoCAP ISAC assays (Thermo Fisher) were performed according to the manufacturer’s instructions. The arrays were visualized by using a LuxScan 10K scanner (Core Life Sciences, Irvine, Calif) and analyzed by using Phadia Microarray Image Analysis software.

### Peanut allergens

For ImmunoCAP inhibition and the mast cell activation test (MAT), the peanut allergens Ara h 2 and Ara h 6 were purchased from Indoor Biotechnologies (Cardiff, United Kingdom). Both Ara h 2 and Ara h 6 were purified natively from peanut kernels by using a combination of affinity and high-performance liquid chromotography. Neither allergen showed contamination with any other peanut allergens (Ara h 1, Ara h 2, Ara h 3, Ara h 6, and Ara h 8), and contamination with each other was minimal (≤0.0005% possible contamination of Ara h 2 with Ara h 6 and possible contamination ≤0.007% of Ara h 6 with Ara h 2 were reported) (see Table E1 in this article’s Online Repository at www.jacionline.org).

### ImmunoCAP inhibition

ImmunoCAP inhibition assays were performed to establish the dominant allergen in Ara h 2– and Ara h 6–dual-sensitized individuals. The samples used in Ara h 2 inhibition assays were selected according to the following criteria: dual sensitization to Ara h 2 and Ara h 6, Ara h 2–specific IgE of >40 kU_A_/L, and sufficient sample volume. Of these 15 samples, 10 were available in volumes sufficient for subsequent Ara h 6 inhibition assays. Before assay, 75 μL of patient sera were incubated for 1 hour at room temperature with 75 μL of 1 μg/mL of native Ara h 2 or 1 μg/mL of native Ara h 6 (both from Indoor Biotechnologies, Cardiff) or PBS. Patient plasma, which was reported to have a level of Ara h 2–specific IgE greater than 200 kU_A_/L (n = 2) in prior analyses, required an initial dilution by half in PBS before the aforementioned allergen/PBS preincubation step to ensure that results would be reported within the assays' measuring range. Preincubated samples were then assayed for Ara h2– and Ara h 6–specific IgE by using ImmunoCAP technology, as already described. Results were expressed as specific IgE concentrations (kU_A_/L) alongside the uninhibited (PBS) control.

### MAT

LAD2 cells, a human mast cell line that was originally isolated from the marrow of a patient suffering with mastocytosis and is named after the National Insitute of Health’s Laboratory of Allergic Diseases,^19^ were cultured in recombinant IL-4 for 5 days before overnight sensitization with patient’s plasma, as previously described.^20^^,^^21^ Serial dilutions (0.1-1000 ng/mL) of the native peanut allergens Ara h 2 or Ara h 6 (Indoor Biotechnologies) or peanut extract were added to sensitized cells and incubated for 1 hour at 37˚C to induce stimulation. Cells were then assessed for surface expression of CD63 by flow cytometry. Anti-IgE (1 μg/mL) and ionomycin (1 μg/mL) were used as IgE-mediated and non–IgE-mediated positive controls, respectively, and 0.04% BSA RPMI medium alone was used as a negative control. Flow cytometry was performed on a FACS Canto II with FACSDiva software (BD Biosciences), and the data were analyzed by using FlowJo software, version 7.6.1 (TreeStar).

### Statistical analysis

Comparisons of unpaired and paired continuous variables were undertaken by using the Mann-Whitney *U* test and Kruskal-Wallis *P* test, respectively. The diagnostic performance of specific IgE to peanut extract and to allergen components was examined against the allergic status to peanut by using receiver operating characteristic (ROC) curve analyses. Optimal cutoff values were generated by determining the Youden index as follows: Sensitivity – (1 – Specificity). Statistical analyses were performed by using GraphPad Prism 7.0 (GraphPad Software, Inc, San Diego, Calif) and SPSS 25.0 (IBM Inc, Armonk, NY) software.

## Results

### Study participants

A total of 100 children (50 with peanut allergy, 40 who were sensitized to but tolerant of peanut, and 10 who were neither sensitized nor allergic to peanut) were included in this study; they ranged in age from 5 months to 17 years, and 69 of them were male. The diagnosis of peanut allergy and determination of sensitization were carried out according to the criteria previously described.^22^ The children with peanut allergy were slightly older than the children who were sensitized to but tolerant of peanut, and they had larger wheals on the SPT and higher levels of specific IgE to peanut Ara h 2, Ara h 3, and Ara h 6, with a large overlap between the 2 groups. Total IgE level was higher in the group of those who were sensitized to but tolerant of peanut. The demographic, clinical, and serologic features of the study population are shown in Table I.

**Table I:** Demographic and clinical features and serum-specific IgE levels of the study population (N = 100)

Characteristic	With peanut allergy (n = 50)	Sensitized to but tolerant of peanut (n = 40)	Not allergic to peanut (n = 10)	*P* value
Age (y)	8.31 (1.68-17.91)	5.93 (0.52-15.81)	5.96 (0.77-12.58)	.003
Males	36 (72%)	25 (62.5%)	8 (80%)	.371
Result of SPT to peanut (mm)	9 (1-34)∗	3 (0-12)∗	0 (0-0)	<.0001
Total IgE level (kU_A_/L)	297.5 (4-3550)	469 (7-10714)	29.5 (6-397)	.5757
Level of specific IgE to peanut (kU_A_/L)	12 (0.2-568)	2.41 (0.04-128)	0.01 (0-0.9)	.0019
Level of specific IgE to Ara h 1 (kU_A_/L)	0.8 (0-199)	0.11 (0-88.3)	0 (0-0.01)	.0838
Level of specific IgE to Ara h 2 (kU_A_/L)	2.54 (0.01-278)	0.07 (0-82.3)	0.04 (0.01-0.07)	<.0001
Level of specific IgE to Ara h 3 (kU_A_/L)	0.36 (0-89.6)	0.06 (0-7.28)	0.01 (0-0.04)	.0456
Level of specific IgE to Ara h 6 (kU_A_/L)	3.835 (0-155)	0.03 (0-86)	0(0-0.02)	<.0001
Level of specific IgE to Ara h 8 (kU_A_/L)	0.09 (0-185)	0.04 (0-88.6)	0 (0-0.02)	.4022
Level of specific IgE to Ara h 9 (kU_A_/L)	0.02 (0-871)	0.05 (0-43.9)	0.01 (0-0.02)	.0834

Values are expressed as number (%) or median (range). *P* value refers to the comparison between patients with peanut allergy and patients sensitized to but tolerant of peanut using Mann-Whitney *U* test.

∗ SPT result for 49 children with peanut allergy and 38 children who were sensitized to but tolerant of peanut.

### Specific IgE to Ara h 2 and Ara h 6 are the best serologic markers to diagnose peanut allergy

**Fig 1:**
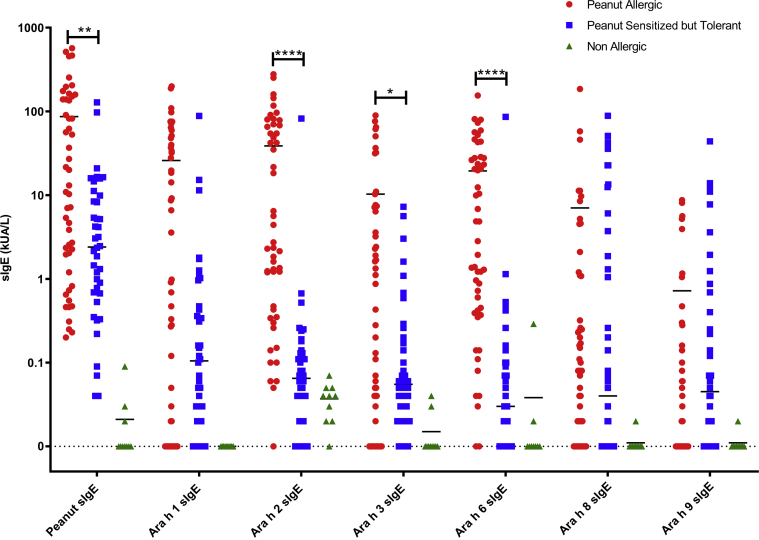
Distribution of specific IgE (sIgE) levels in individuals with peanut allergy (n = 50), individuals sensitized to but tolerant of peanut (n = 40), and individuals neither sensitized nor allergic to peanut (n = 10). The Mann-Whitney *U* test was used for the comparison between individuals with peanut allergy and individuals sensitized to but tolerant of peanut. ∗*P* < .05; ∗∗*P* < .01; ∗∗∗*P* < .001; ∗∗∗∗*P* < .0001.

Levels of specific IgE to peanut (*P* = .019), Ara h 2 (*P* < .001), Ara h 3 (*P* = .046), and Ara h 6 (*P* < .001) were all significantly higher in children with peanut allergy than in individuals sensitized to but tolerant of peanut. Conversely, Ara h 1–, Ara h 8–, and Ara h 9–specific IgE were not able to discriminate between those individuals in this population who had peanut allergy and those who were sensitized to but tolerant of peanut (Fig 1).

**Fig 2:**
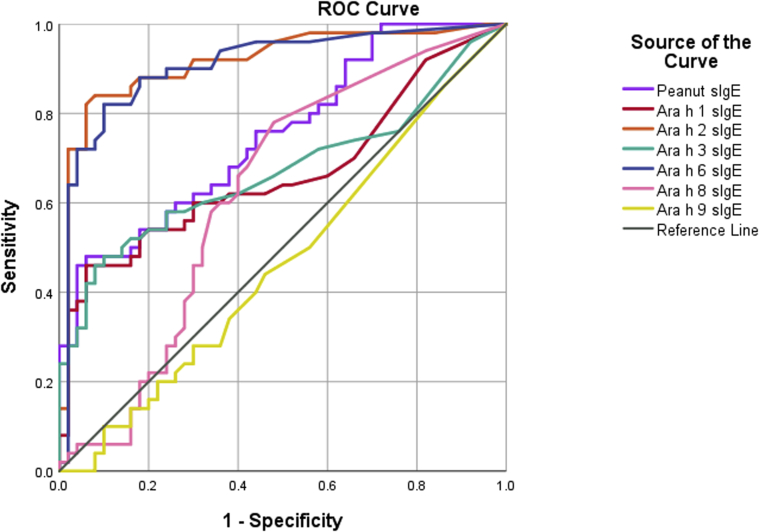
ROC curves for IgE specific to peanut (*purple*), Ara h 1 (*red*), Ara h 2 (*orange*), Ara h 3 (*green*), Ara h 6 (*blue*), Ara h 8 (*pink*), and Ara h 9 (*yellow*) across the entire study population (N = 100). For areas under the ROC curves and the respective 95% CIs, see Table E2. *sIgE*, Specific IgE.

The diagnostic utility of each serologic marker was assessed by using ROC curve analyses (Fig 2). The areas under the ROC curve for Ara h 2–specific IgE (0.916) and Ara h 6–specific IgE (0.908) were far greater than the area under the ROC curve for IgE to peanut or any of the other allergen specificities assessed (see Table E2 in this article’s Online Repository at www.jacionline.org). Following the initial analyses, optimal cutoffs for Ara h 2–specific IgE (0.28 kU_A_/L) and Ara h 6–specific IgE (0.32 kU_A_/L) were generated. They both reported 82% sensitivity, but the cutoff for Ara h 2–specific IgE had 94% specificity to diagnose peanut allergy and the cutoff for Ara h 6–specific IgE had 90% specificity.

### The majority of patients with peanut allergy are cosensitized to Ara h 2 and Ara h 6

**Fig 3:**
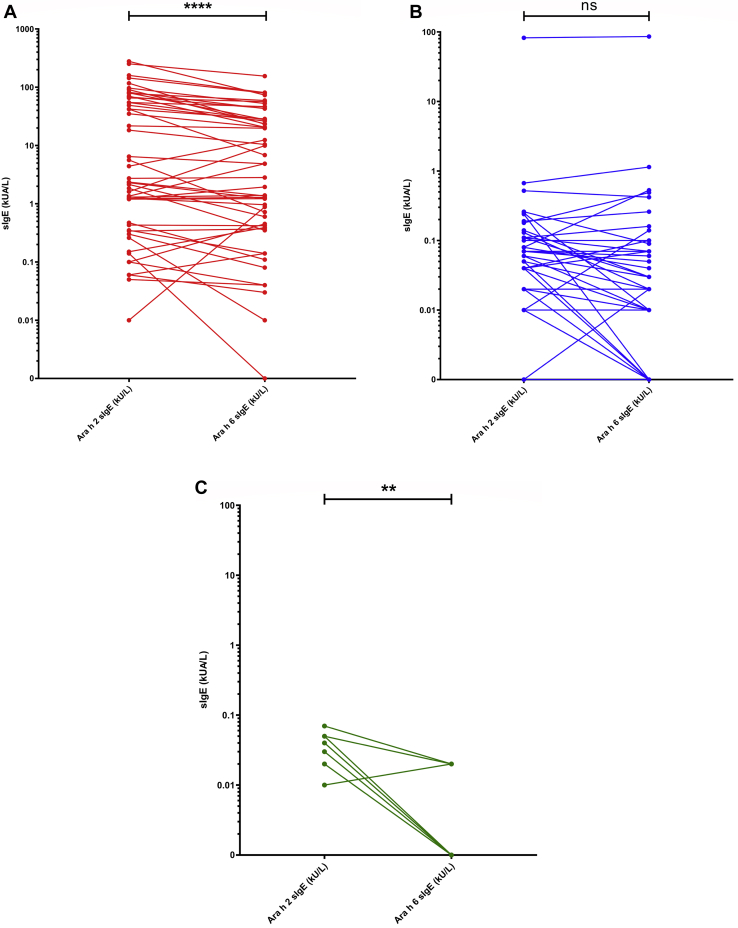
Paired Ara h 2–specific IgE and Ara h 6–specific IgE levels across individuals with peanut allergy (**A**) (n = 50; *P* < .001), individuals sensitized to but tolerant of peanut (**B**) (n=40; *P* = .240), and individuals neither sensitized nor allergic to peanut (**C**) (n = 10; *P* = .004). The Wilcoxon signed rank test was used for the comparison between Ara h 2–specific and Ara h 6–specific IgE levels, ∗*P* < .05; ∗∗*P* < .01; ∗∗∗ *P* < .001; ∗∗∗∗ *P* < .0001. *ns*, Not significant; *sIgE*, specific IgE.

**Fig 4:**
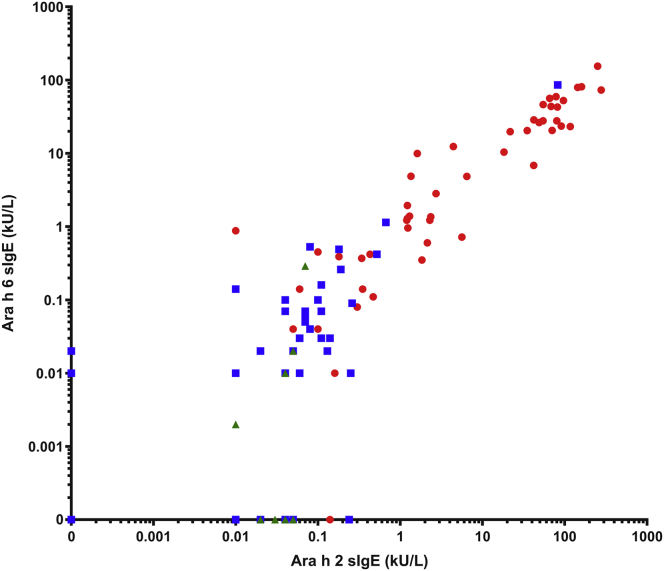
Correlation between specific IgE (sIgE) to Ara h 2 and Ara h 6 across the whole study population; individuals with peanut allergy (n = 50 [*red*]); individuals sensitized to but tolerant of peanut (n = 40 [*blue*]); and individuals who were neither allergic nor sensitized to peanut (n = 10 [*green*]). Spearman correlation test; *R* = 0.8791.

**Fig E1:**
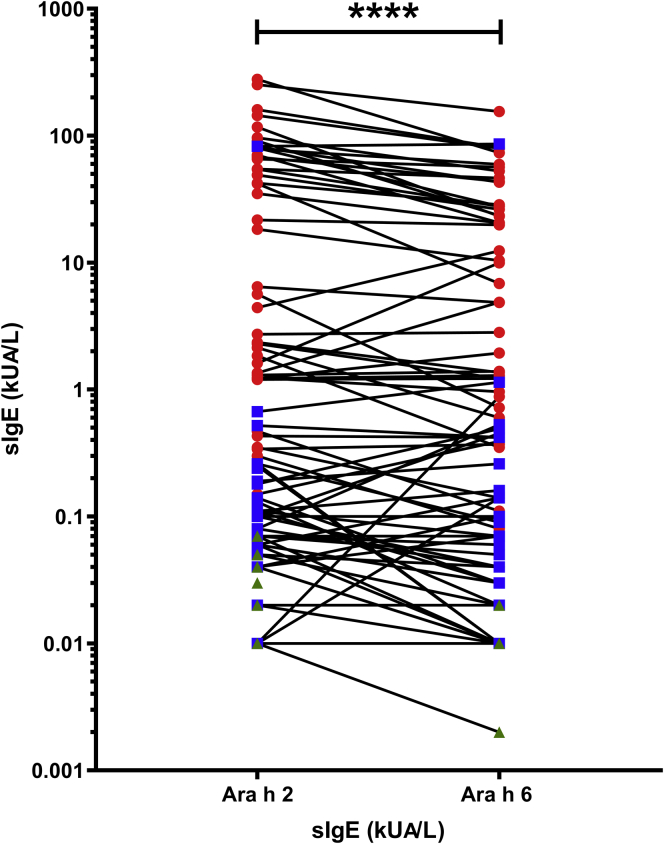
Paired Ara h 2– and Ara h 6–specific IgE (sIgE) levels across the whole study population (N = 100). The Wilcoxon signed rank test was used for the comparison between Ara h 2–and Ara h 6– specific IgE levels (*P* < .0001). *sIgE*, Specific IgE.

Considering the similarities in sequence and structure between Ara h 2 and Ara h 6 molecules, we wanted to assess which of the 2 allergens was dominant in our patient population. Most individuals sensitized to peanut were sensitized to both Ara h 2 and Ara h 6. For instance, Ara h 2 and Ara h 6 sensitization was seen in 61 of 90 individuals (68%) and 55 of 90 individuals (61%) sensitized to peanut, respectively, with 49 of 90 individuals (54%) reporting Ara h 2 and Ara h 6 cosensitization. As shown in Fig 3, *A-C*, in the group of children sensitized to but tolerant of peanut, there was no significant difference between Ara h 2–specific IgE and Ara h 6–specific IgE levels (*P* = .084). However, for the group of those with peanut allergy, the levels of Ara h 2–specific IgE were significantly higher than the levels of Ara h 6–specific IgE (*P* < .001), suggesting Ara h 2 as the dominant allergen. This skewing of IgE sensitization toward Ara h 2 also appears to be true across the whole study population, in which the significant difference remains (*P* < .001) (see Fig E1 in this article’s Online Repository at www.jacionline.org). Despite this, a strong correlation was observed between Ara h 2–specific IgE and Ara h 6–specific IgE levels (*r* = 0.8791), as seen in Fig 4.

In this study, 2 individuals (4%) from the group with peanut allergy (n = 50) showed detectable levels of specific IgE to Ara h 6 (≥0.1) but remained unsensitized for Ara h 2 (<0.1) (see Table E3 in this article’s Online Repository at www.jacionline.org). Of these, only 1 individual (patient 164) reported genuine monosensitization to Ara h 6 (ie, this individual did not have detectable IgE for any of the other peanut components [considering singleplex ImmunoCAP]). Peanut allergy in this patient was confirmed by OFC. Subsequent investigations of this patient’s sample led to ImmunoCAP ISAC analyses, which, however, indicated sensitization to both Ara h 2 (0.6 ISAC standardized units) and Ara h 6 (0.8 ISAC standardized units) but not to any of the other 110 allergens present in that assay.

### IgE of Ara h 2– and Ara h 6–dual-sensitized individuals predominantly binds Ara h 2

Of the 50 individuals with peanut allergy who were included in this study, 42 (84%) were sensitized to both Ara h 2 and Ara h 6, with 31 of 42 (74%) reporting higher levels of Ara h 2–specific IgE than Ara h 6–specific IgE. To test for possible cross-reactivity between Ara h 2 and Ara h 6 binding, we performed ImmunoCAP inhibition experiments examining binding to Ara h 2 and Ara h 6 by using samples from 15 dual-sensitized individuals with peanut allergy (Table II). In these experiments, IgE binding to both Ara h 2 and Ara h 6 was assessed following preincubation of sera, in a 1:1 ratio, with PBS, Ara h 2, or Ara h 6.

**Table II:** Demographic and serologic features of individuals whose samples were used in Ara h 2 inhibition (n = 15), Ara h 6 inhibition∗ (n = 10), and MAT∗ (n = 10) experiments

Patient ID	Age (y)	Sex	Peanut SPT	Total IgE (kU_A_/L)	Peanut-specific IgE (kU_A_/L)	Ara h 1–specific IgE (kU_A_/L)	Ara h 2–specific IgE (kU_A_/L)	Ara h 3–specific IgE (kU_A_/L)	Ara h 6–specific IgE (kU_A_/L)	Ara h 8–specific IgE (kU_A_/L)	Ara h 9–specific IgE (kU_A_/L)
018	6.95	F	20	1872	466	109	144	7.41	79.5	0.07	1.05
020∗	15.93	F	34	542	90.5	48	80	10.3	27.8	8.47	0.01
041	17.91	F	10	430	159	36.9	96.5	2.41	52.6	0.01	0.01
083∗	6.45	M	14	799	163	27.9	65.5	32.3	56.4	0.11	0.47
090∗	5.94	M	12	928	135	71.7	54.4	1.33	27.8	0.08	0.08
096∗	15.91	M	22	328	139	39.9	68.1	3.59	43.6	0.24	0
101∗	4.22	M	10	76	62.1	18.5	42	0.2	6.84	0	0
103∗	16.90	M	7	906	82.1	3.57	54.6	7.62	46.4	11.3	0.14
108	8.97	M	7	874	254	59.5	117	61.5	23.2	0.32	0.04
112∗	14.54	M	14	823	197	33.5	90.7	31.6	23.5	4.6	0.3
119∗	8.33	M	10	3531	568	199	278	89.6	73.2	0.1	0.1
122	6.01	M	13	545	175	51.2	78.4	11	59.3	4.53	5.62
125∗	10.38	M	14	3383	515	189	160	76.1	80.9	0.17	1.16
134	11.50	F	19	496	205	97.7	70.3	36.6	20.6	0.15	0.06
184∗	7.74	F	4	1796	455	75.4	252	64.8	155	0.05	0.06

*F*, Female; *ID*, identifier; *M*, male.

∗ Peanut allergic patients tested on Ara h 6 inhibition assay and the mast cell activation tests to Ara h 2 and Ara h 6.

**Fig 5:**
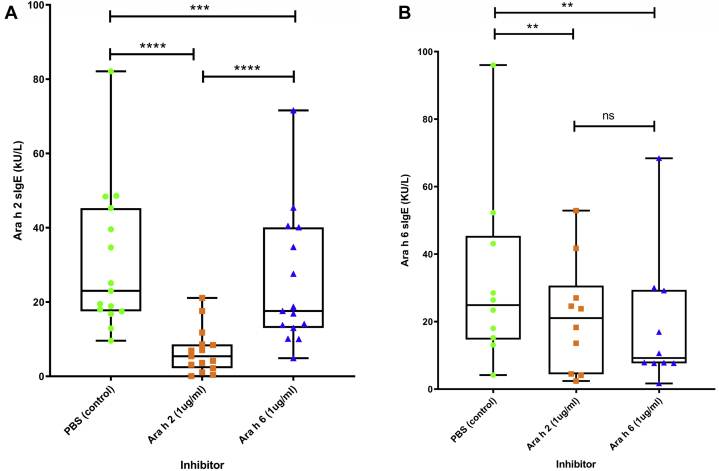
Inhibition of IgE binding to Ara h 2 **(A)** and Ara h 6 **(B)** following preincubation of serum with PBS (control), Ara h 2, or Ara h 6. The Wilcoxon signed rank test was used for the comparison between bound Ara h 2–specific IgE (sIgE) and Ara h 6–specific IgE. ∗*P* < .05; ∗∗*P* < .01; ∗∗∗*P* < .001; ∗∗∗∗*P* < .0001. *sIgE*, Specific IgE.

Inhibition of Ara h 2 binding assays demonstrated that preincubation of sera with Ara h 2 (*P* < .001) or Ara h 6 (*P* < .001) was able to significantly block binding of IgE to Ara h 2, in comparison with the PBS controls (Fig 5, *A*). Notably, Ara h 2 preincubation led to a significantly greater inhibition of Ara h 2 binding than Ara h 6 (*P* < .001). Ara h 6 binding following preincubation with either Ara h 2 (*P* = .002) and Ara h 6 (*P* = .002) showed significant inhibition (Fig 5, *B*). However, preincubation with Ara h 2 inhibited IgE binding to the Ara h 6 ImmunoCAP to a degree similar to that with preincubation with Ara h 6. These data suggest that Ara h 2 is the dominant allergen in Ara h 2– and Ara h 6–dual-sensitized individuals.

### Ara h 2 induces greater effector cell activation than Ara h 6 in Ara h 2– and Ara h 6–dual-sensitized individuals with peanut allergy

To relate Ara h 2 and Ara h 6 IgE binding to functional effector cell activation following stimulation with allergen, we took the same 10 dual-sensitized individuals with peanut allergy as assessed in the Ara h 2 and Ara h 6 inhibition experiments and performed MAT assays against both Ara h 2 and Ara h 6 across a range of concentrations.

**Fig 6:**
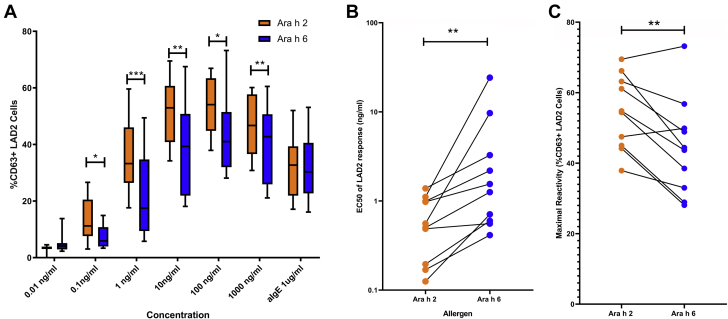
**A,** Results of the MAT to both Ara h 2 and Ara h 6 in children with peanut allergy (n = 10). Increasing concentrations of allergen from 0.01 ng/mL to 1000 ng/mL and 1 μg/mL of anti-IgE were used to stimulate LAD2 cells previously sensitized with the patient’s plasma. A paired *t* test was used to compare the percentage of CD63^+^ LAD2 cells between Ara h 2 and Ara h 6. **B,** Comparison of LAD2 (percentage of CD63 cells) EC_50_ in response to Ara h 2 and Ara h 6 stimulation in dual-sensitized patients (*P* = .002 with the Wilcoxon signed rank test). **C,** Comparison of paired maximal reactivity in response to Ara h 2 and Ara h 6 stimulation in dual-sensitized patients (*P* < .01 with the Wilcoxon signed rank test). ∗*P* < .05; ∗∗*P* < .01; ∗∗∗*P* < .001; ∗∗∗∗*P* < .0001. *sIgE*, Specific IgE.

**Fig E2:**
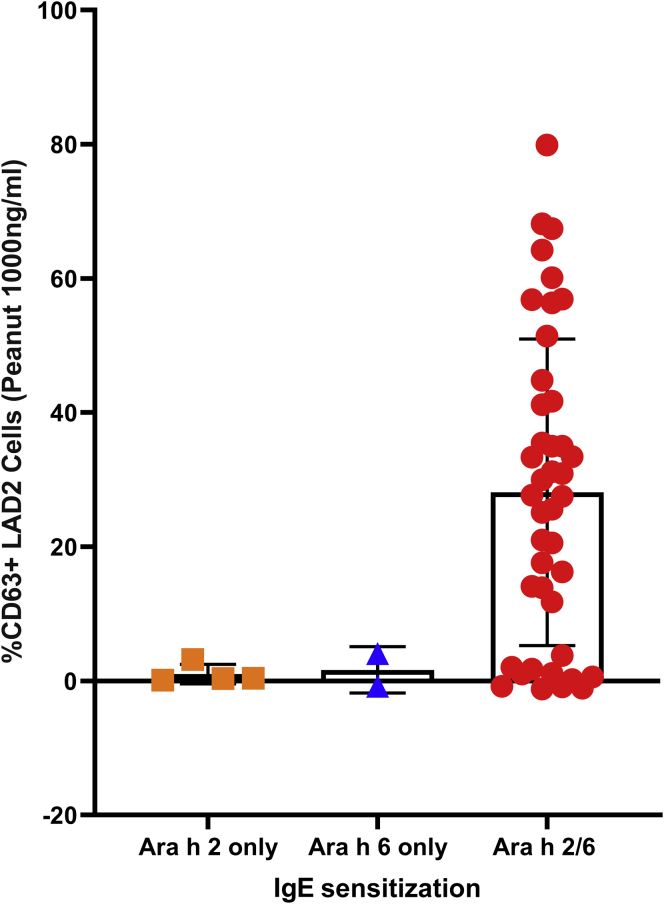
Results of the MAT to peanut in individuals with peanut allergy (n = 48). Individuals were stratified by monosensitization to Ara h 2 or Ara h 6 or cosensitization to both allergens.

MAT analyses showed that Ara h 2 was able to induce a significantly greater proportion of activated mast cells (defined as percentage of CD63^+^ LAD2 cells in relation to the negative control) than Ara h 6 at 0.1 ng/mL (*P* = .033), 1 ng/mL (*P* < .001), 10 ng/mL (*P* = .005), 100 ng/mL (*P* = .013), and 1000 ng/mL (*P* < .001) of allergen (see Fig 6, *A*). Sensitivity and reactivity of the LAD2 cells in response to both allergens were also assessed. All half maximal effective concentration (EC50) values were calculated for both Ara h 2 and Ara h 6 dose responses, with Ara h 6 stimulation showing a significantly greater EC50 (*P* = .002) (see Fig 6, *B*). The maximal reactivity (greatest percentage of CD63^+^ activation) was significantly higher for Ara h 2 than for Ara h 6 in these dual-sensitized individuals (*P* < .01) (see Fig 6, *C*). Cosensitization to Ara h 2 and Ara h 6 was associated with a higher proportion of activated mast cells (see Fig E2 in this article’s Online Repository at www.jacionline.org).

## Discussion

Ara h 2 and Ara h 6 share sequence and structural similarities, and the majority of patients with peanut allergy are sensitized to Ara h 2. In this study, we confirmed that Ara h 6 is also a major peanut allergen in our patient population. Although most patients were cosensitized to both Ara h 2 and Ara h 6, levels of Ara h 6–specific IgE were generally lower in patients with peanut allergy, and the degree of IgE inhibition to both allergens was higher following preincubation with Ara h 2 than with Ara h 6, suggesting that Ara h 2 is the dominant allergen. Ara h 2 was a more efficient inducer of effector cell activation than Ara h 6 was. We evaluated the diagnostic utility of specific IgE to Ara h 6 in a large cohort of children being assessed for peanut allergy and generated optimal cutoffs for both Ara 6 and Ara h 2 that can be reliably used in the United Kingdom and other areas with similar populations of patients with peanut allergy. A level of specific IgE to Ara h 6 greater or equal than 0.32 kU_A_/L confirms peanut allergy with 82% sensitivity and 90% specificity, with Ara h 2–specific IgE greater or equal to 0.26 kU_A_/L improving specificity slightly (94%) with the exact same sensitivity; thus, Ara h 2–specific IgE captures more cases with peanut allergy, and Ara h 6–specific IgE is rarely needed as an additional test for peanut allergy.

To our knowledge, this is the largest study assessing the diagnostic performance of Ara h 6–specific IgE in the singleplex ImmunoCAP assay and the first in the United Kingdom. Ara h 2 has long been considered the best marker for peanut allergy in both adult and pediatric patients, and this was reflected in our population^.^^7^^,^^8^^,^^23^ Ara h 6, most likely because of its sequential and structural similarity, has been reported alongside Ara h 2 as an important marker for peanut allergy, both serologically and as an inducer of effector cell response.^9^, ^10^, ^11^^,^^16^^,^^24^ In our cohort, we confirmed that Ara h 6 is a major allergen in the United Kingdom. However, the higher levels of Ara h 2–specific IgE and the greater IgE-inhibitory capacity of Ara h 2 suggest Ara h 2 dominance when compared with Ara h 6. The majority of our patients with peanut allergy (84%) were cosensitized to both Ara h 2 and Ara h 6, and only a minority were monosensitized to either Ara h 6 (4%) or Ara h 2 (8%). A case series recently published by van der Valk et al highlighted the clinical importance, albeit uncommonness, of monosensitization to Ara h 6 and related it to moderate to severe allergic reactions to peanut.^25^ Only 1 individual with peanut allergy in our cohort was monosensitized to Ara h 6 on the basis of initial ImmunoCAP testing; however, genuine monosensitization is dubitable following subsequent ImmunoCAP ISAC analyses that reported sensitization to both Ara h 2 and Ara h 6, with higher levels of Ara h 6–specific IgE than Ara h 2–specific IgE. Despite the generally low levels of specific IgE, an OFC was performed in this individual and confirmed peanut allergy. In this instance, another characteristic of IgE, such as IgE affinity, as opposed to titer and specificity, may be more influential in triggering the allergic response by mast cells and basophils.

Identifying the dominant 2S albumin between Ara h 2 and Ara h 6 is always likely to be complicated by the structural and/or binding similarities, with 41 shared epitopes previously identified between the 2 allergens.^26^ However, the Ara h 2 and Ara h 6 allergens used had minimal, if any, contamination with each other. Herein, we adapted methods of IgE inhibition from Amoah et al to test our hypotheses.^27^ However, in contrast to that study, we utilized the technique on an individual patient basis rather than pooled sera. This is the first data of its type assessing Ara h 2 and Ara h 6 competition in individuals with peanut allergy. The incorporation of ImmunoCAP technology into the inhibition experiments allowed for fully quantitative IgE measurements, highly sensitive detection of changes in specific IgE concentration, and also reproducibility in other patient populations, which is a particular strength of the ImmunoCAP technology. We noted, however, some technical variation in the levels with samples being selected on the basis of having levels higher than 40 kU_A_/L and showing (in a 1:1 dilution) levels below 20 kU_A_/L, but the majority of these samples showed levels very close to 20 kU_A_/L after dilution, and this variation would not have affected the results, as inhibition was expressed in relation to the respective PBS control, which was tested simultaneously. Inhibition experiments revealed Ara h 2 as the dominant inhibitory allergen, demonstrating an ability to reduce IgE binding to both Ara h 2 and Ara h 6 to a significantly greater degree than Ara h 6 did. This is consistent with previous data from our own laboratory and with data published by Bernard et al, in which additional linear epitope(s) that were identified on native Ara h 2, and not conserved within native Ara h 6, may offer unique binding sites.^28^^,^^29^ The additional linear epitope(s) on Ara h 2 is likely to increase the binding of Ara h 2–specific IgE. Notably, however, both Ara h 2 and Ara h 6 were able to inhibit IgE binding to each other, suggesting that Ara h 2– and Ara h 6–specific IgE in patients with peanut allergy is a mix of IgE resulting from primary sensitization and cross-reactivity, concordant with data recently published by Hazebrouck et al.^30^ Both these data and our results presented here suggest variability among individuals, with different proportions of IgE antibodies binding uniquely or cross-reactively to Ara h 2 and Ara h 6. These observations led us to perform functional MATs in the dual-sensitized individuals for whom both Ara h 2 and Ara h 6 inhibition experiments were undertaken. Similarly, Ara h 2 induced activation to a greater degree in these individuals, even though both allergens were able to generate a substantial response. The distinctive biologic activity can be better highlighted through comparison of both the EC50 values and maximal reactivity. Ara h 2 dose responses generated significantly lower EC50 values, but significantly greater maximum reactivity, in the same dual-sensitized individuals. In other words, lower concentrations of Ara h 2 were needed to cause similar effector cell activation, with Ara h 2 inducing a larger proportion of activated mast cells than Ara h 6 did. Monosensitization to either Ara h 2 or Ara h 6 was associated with a lower degree of mast cell activation to peanut than was cosensitization to Ara h 2 and Ara h 6. Given that greater mast cell activation is associated with more severe reactions, being cosensitized to both allergens could convey a higher risk of anaphylaxis.^21^

Overall, our data showed that independent as well as cross-reactive sensitizations to Ara h 2 and Ara h 6 are present in dual-sensitized individuals but indicated that Ara h 2 is likely the dominant allergen in our population of United Kingdom–based children. Hence, we consider Ara h 2–specific IgE the most important diagnostic marker in peanut allergy even if Ara h 6–specific IgE levels may need to be determined in rare individual cases. As well as confirming Ara h 2 as the most dominant peanut allergen and demonstrating its superior diagnostic accuracy, our study may inform future allergen-specific immunotherapy (AIT) and other immunomodulatory treatments. Peanut AIT has shown potential as disease-modifying therapy, with large numbers of individuals undergoing treatment reporting reduced symptoms and increased levels of peanut-specific IgG4.^31^^,^^32^ Being the most important elicitors of peanut allergic reactions, Ara h 2 and Ara h 6 may also be the key peanut allergens to use for AIT. Although immunotherapy can induce changes in levels of IgE and IgG_4_ to different peanut allergens, a recent study showed that changes in antibody titer in response to peanut AIT were predominantly to Ara h 2– and Ara h 6–specific IgE and IgG_4_, with significant increases in Ara h 2 and Ara h 6 IgG_4_/IgE ratios, highlighting the importance of these 2 allergens in peanut allergy, which is consistent with our findings.^33^, ^34^, ^35^, ^36^ We believe that if food AIT, like diagnostics, becomes more targeted to specific allergens over the coming years, the best candidates for peanut AIT could be Ara h 2 and Ara h 6. This is concordant with previous data generated in a study by Kulis et al, in which a combinatory Ara h 2 and Ara h 6 immunotherapy protocol was able to significantly reduce symptoms and functional cell activation in allergic mouse models.^37^ Despite our data suggesting that Ara h 2 alone would be sufficient to diagnose peanut allergy in the majority of individuals, the presence of both independent and cross-reactive Ara h 2– and Ara h 6–specific IgE antibodies in most individuals with peanut allergy illustrates the potential benefit of combinatory Ara h 2 and Ara h 6 therapy.

**Clinical implications**

Ara h 2 is the dominant allergen and a more potent elicitor of mast cell activation than Ara h 6 is. Specific IgE to Ara h 2 is sufficient to diagnose peanut allergy in the majority of patients.

## Figures and Tables

**Table E1:** Percentage contamination of Ara h 2 and Ara h 6 with other peanut allergens

Allergen	Contamination (%)				
	Ara h 1	Ara h 2	Ara h 3	Ara h 6	Ara h 8
Ara h 2	0	—	0.007	0.0005	0.0003
Ara h 6	0	0.007	0	—	0

Data are as provided and performed by Indoor Biotechnologies as part of the quality control procedure. Results have been generated by using ELISA.

**Table E2:** Performance of specific IgE to peanut and individual peanut allergens in the diagnosis of peanut allergy, as governed by area’s under the Receiver Operator Characteristic curve (AUC)

Marker	AUC	95% CI	Cutoff (kU_A_/L)	Sensitivity (%)	Specificity (%)	PPV (%)	NPV (%)
Peanut-specific IgE	0.752	0.658-0.845	21.25	32.29	93.1	92	36
Ara h 1-specific IgE	0.668	0.559-0.777	2.68	32.86	90	88.46	36.49
Ara h 2-specific IgE	0.916	0.857-0.975	0.28	82	94	93.18	83.93
Ara h 3-specific IgE	0.672	0.563-0.78	1.1	33.33	87.1	85.19	36.99
Ara h 6-specific IgE	0.908	0.846-0.97	0.32	82	90	89.13	83.33
Ara h 8-specific IgE	0.624	0.511-0.737	0.015	60	31.43	61.9	29.73
Ara h 9-specific IgE	0.468	0.354-0.581	0.86	14.58	82.69	43.75	51.19

*AUC*, Area under the curve; *NPV*, negative predictive value; *PPV*, positive predictive value.

**Table E3:** Demographic and serologic features of individuals with peanut allergy and reporting sensitization to Ara h 6 but not to Ara h 2

Patient ID	Age (y)	Sex	Peanut SPT result (mm)	Total IgE level (kU_A_/L)	Peanut sIgE level (kU_A_/L)	Ara h 1 sIgE level (kU_A_/L)	Ara h 2 sIgE level (kU_A_/L)	Ara h 3 sIgE level (kU_A_/L)	Ara h 6 sIgE level (kU_A_/L)	Ara h 8 sIgE level (kU_A_/L)	Ara h 9 sIgE level (kU_A_/L)
087	5.78	M	6	540	2.7	0.02	0.06	0.11	0.14	1.2	8.1
164	1.68	M	4	6.36	0.46	0.01	0.01	0.01	0.88	0.02	0.01

*ID*, Identifier; *M*, male; *sIgE*, specific IgE.
